# Esophagogastric polyurethane bezoar complicated by stomach wall microperforation and acute peritonitis: case report

**DOI:** 10.1186/s12893-020-00945-y

**Published:** 2020-11-12

**Authors:** Krzysztof Ziaja, Jerzy Chudek, Aleksander Chlubek, Mariola Sznapka, Tomasz Toborek, Damian Ziaja

**Affiliations:** 1 Department of Oncological Surgery with the Subunit of Vascular Surgery, Oncological Centre in Katowice, Katowice, Poland; 2 Department of General Surgery, District Hospital in Wodzisław Śląski, Wodzisław Śląski, Poland; 3 Department of Internal Diseases and Oncological Chemotherapy, Faculty of Medical Sciences in Katowice, Medical University of Silesia, Katowice, Poland; 4 Górnośląska Wyższa Szkoła Handlowa Wydział Medyczny, Katowice, Poland; 5 Department of Physiotherapy, Faculty of Health Sciences in Katowice, Medical University of Silesia, Katowice, Poland

**Keywords:** Polyurethane bezoars, Stomach perforation, Acute peritonitis, Case report

## Abstract

**Background:**

Bezoars are collections of indigestible material in the gastrointestinal tract, mostly described in children. Polyurethane “plastobezoars” consisting of composites used in the construction industry are rarely described bezoars formed in the esophagus and stomach, causing gastrointestinal obstruction, usually necessitating gastrectomy.

We describe an unusual presentation of polyurethane bezoar with a volcanic rock consistency, that caused gastrointestinal obstruction and perforation of the stomach wall.

**Case presentation:**

A 39-year-old man, a construction worker, was referred with signs and symptoms of high gastrointestinal obstruction and abdominal pain. Esophagoscopy revealed a foreign body in the esophagus, 20 cm from the incisor line, causing its obstruction. The attempt to collect the material with forceps failed as the material was too hard. Spiral computed tomography visualized a wide, gas-filled esophagus and a large stomach. The patient with symptoms of acute peritonitis was operated. There were several microperforations of the stomach wall, caused by sharp bezoar fragments that filled the upper one-third of the stomach and lower part of the esophagus. After a longitudinal stomach incision, the bezoar was bluntly dissected from the wall and removed, and the stomach microperforations were closed by wall duplication. After the operation, the patient confessed to drinking, of his own free will, a two-component building foam used to seal pipes. The patient started normal feeding on the 4th day and was discharge home.

**Conclusions:**

Polyurethane bezoars may cause stomach wall perforation and acute peritonitis. Computed tomography has limited usefulness in patients with polyurethane bezoars due to their low specific weight.

## Background

Bezoars are collections of indigestible material in the gastrointestinal tract, mostly described in children. Current classification of bezoars, divided into four groups: phytobezoars, trichobezoars, lactobezoars, and pharmacobezoars, is incomplete. Since 2011, there have been three published case reports of bezoars consisting of composites used in the construction industry as binders or fillers [1–3]. These polyurethane “plastobezoars” formed in the esophagus and stomach were the cause of obstruction, usually necessitating gastrectomy [2, 3]. Endoscopic removal of bezoar material was reported only by Girardin et al. [1].

We describe a bezoar with consistency of a volcanic rock, arose after the patient drank a two-component polyurethane system for the production of self-expanding foam that caused gastrointestinal obstruction and perforation of the stomach wall.

## Case presentation

A 39-year-old man, a construction worker, without remarkable medical history, was referred to the Department of Surgery with signs and symptoms of high gastrointestinal obstruction (vomiting during meals) and nonspecific abdominal pain. The general conditions were good. There were no dehydration or peritoneal symptoms, and peristaltic sounds were heard by auscultation. The patient reported that symptoms occurred suddenly without apparent reason. Routine laboratory tests revealed mild leukocytosis (12.2 g/L) and increased serum C-reactive protein level (548 mg/L).

An intravenous fluid therapy was started. Stool was obtained after enema, but an attempt to insert the gastric tube failed. Esophagoscopy performed on the 4th day after admission (due to dissimulation and shortage of qualified staff during weekend), revealed a foreign body with a texture similar to a dried building foam in the esophagus, 20 cm from the incisor line, causing its obstruction. The attempt to collect the material with forceps failed as the material was too hard.

**Fig. 1:**
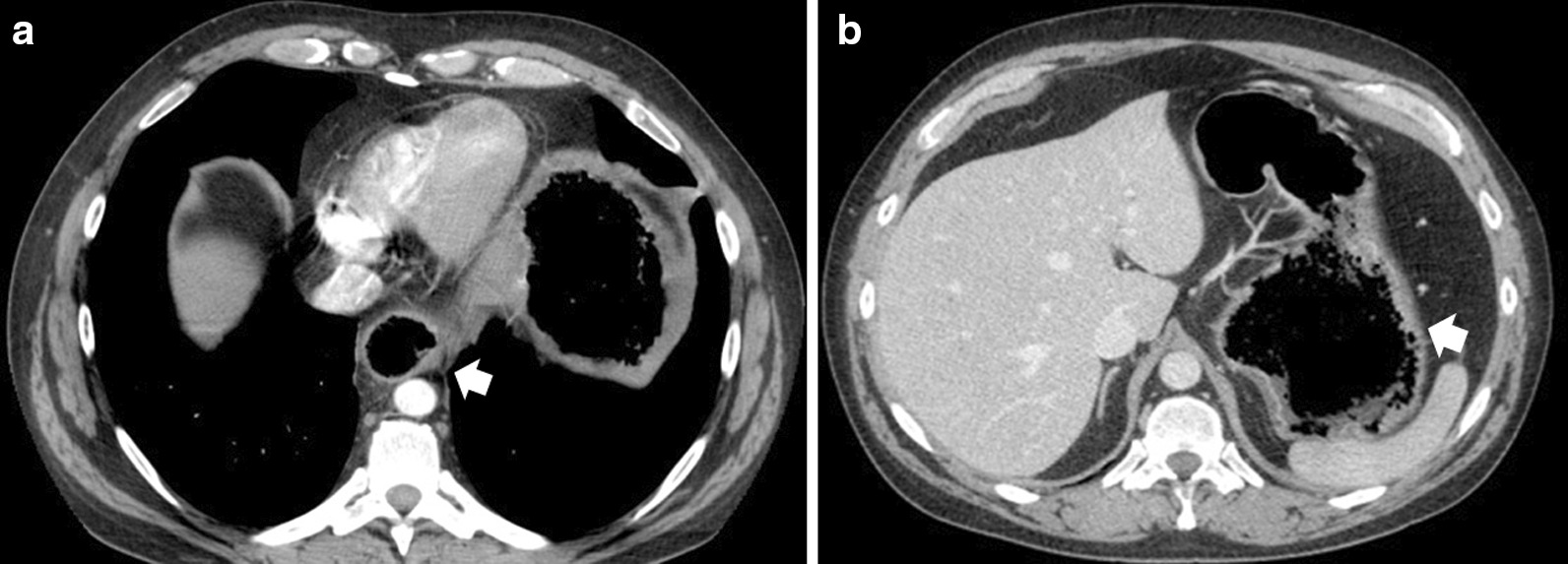
Computed tomography scans showing distension of the esophagus (**a**) and gas-like–filled bottom of the stomach (**a**, **b**)

Spiral computed tomography (CT) visualized only a wide, gas-filled esophagus; a large stomach; and possible hiatal hernia of the diaphragm (Fig. 1).

**Fig. 2:**
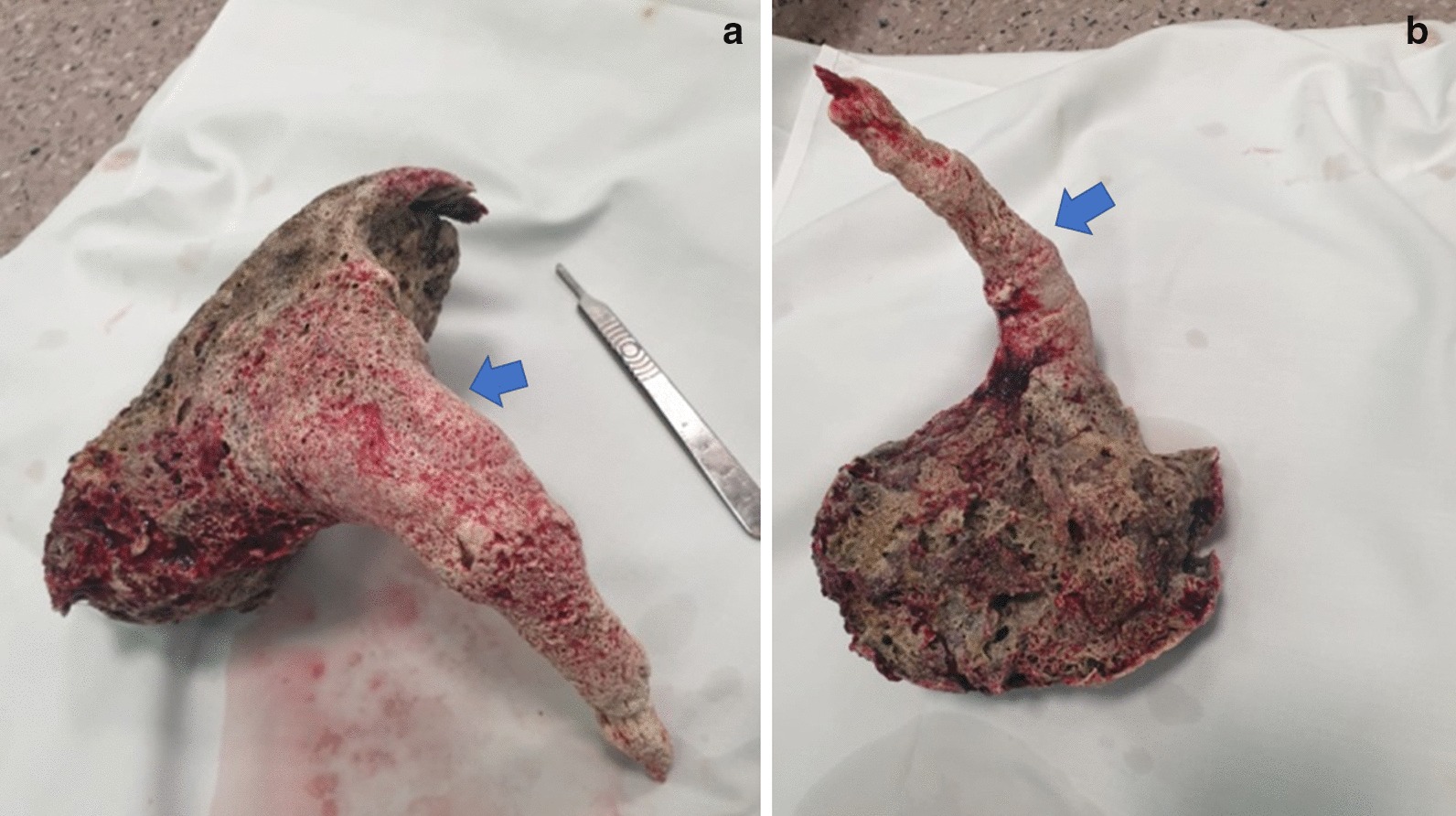
Bezoar removed from the esophagus and stomach. Visible fragments of the esophagus and stomach mucosa ingrown into the bezoar (indicated by arrow). Length was 28 cm (the part in the esophagus, 14 cm); maximum width of the stomach was 12 cm; weight was 374 g; volume was 835 mL (specific weight 0.448 g/cm^3^)

The patient with enhancing abdominal pain and symptoms of acute peritonitis was qualified for a laparotomy (5th day after admission). After peritoneal incision, an outflow of stinking fluid from the left epigastrium was observed. There were several microperforations of the stomach wall, caused by sharp bezoar fragments (of the foreign body) of stone consistency that filled the upper one-third of the stomach and lower part of the esophagus. After a longitudinal stomach incision with surgical electric knife BOWA ARC 400 (50 Watts), the foreign body (bezoar) was bluntly dissected from the stomach and esophagus wall and removed (Fig. 2). The stomach wall microperforations were closed by wall duplication. A feeding tube was passed into the duodenum before the gastric wall incision was closed with a single-layer suture. The abdominal cavity was rinsed with 1 L saline and drained. Before closing the peritoneal cavity, 250 mL of saline stained with methylene blue was injected through the feeding tube; no leakage was found.

After the operation, the patient confessed to drinking, of his own free will, a two-component building foam used to seal pipes. It was established that the only product used in the patient's workplace was a two-component rigid polyurethane foam with increased density, IZY FOAM 100.

Psychiatric consultation indicated only the patient's prolonged difficult life situation.

During the postoperative care, the patient received omeprazole (40 mg twice a day) and antibiotics. We observed rapid improvement in the patient's clinical condition. The patient started normal feeding on the 4th day and was discharge home on the 7th day with the recommendation of psychotherapy and gastroscopic control after 3 months period.

## Discussion and conclusions

The described bezoar arose after the patient drank IZY FOAM 100, a two-component polyurethane system for the production of self-expanding foam. The system consists of polyol and isocyanate components mixed in a ratio of 100:110, which increase volume ten times and reach full mechanical strength after 24 h [4]. The material forming bezoar was very hard, which precluded collection of a sample for testing during gastroscopy. The only way for removal was laparotomy, similarly to the previously described cases [2, 3].

The heat generated during the polymerization reaction between foam components [5] might potentially cause thermal injury to the tissues during direct contact. Therefore, we cannot exclude that this thermal injury and toxicity of isocyanate compound had some role in the formation of microperforations of the stomach wall. In the previously described cases of polyurethane bezoars, no perforations were observed [1–3]. However, such perforations in other bezoars were described as the consequence long-term gastric wall alterations—hyperplastic polyps and deep ulcers due to pressure necrosis.

The specific weight of hardened polyurethane foam was very low, 0.448 g/cm^3^. This is much less than water gravidity and explains the difficulties in interpretation of the CT imaging. In consequence, the distension of the esophagus and stomach was assigned to gas accumulation (tympanites), as the radiologist has no knowledge of previous gastroscopy findings.

Our case demonstrates the risk of stomach wall perforation with development of acute peritonitis and limited usefulness of computed tomography in patients with polyurethane bezoars.

## Data Availability

Not applicable.

## Abbreviations

CT: Computed tomography
IZY FOAM 100: Two-component polyurethane system for the production of self-expanding foam
